# Intracellular Pathways and Mechanisms of Colored Secondary Metabolites in Cancer Therapy

**DOI:** 10.3390/ijms23179943

**Published:** 2022-09-01

**Authors:** Ani-Simona Sevastre, Elena Victoria Manea, Oana Stefana Popescu, Daniela Elise Tache, Suzana Danoiu, Veronica Sfredel, Ligia Gabriela Tataranu, Anica Dricu

**Affiliations:** 1 Department of Pharmaceutical Technology, Faculty of Pharmacy, University of Medicine and Pharmacy of Craiova, Str. Petru Rares nr. 2-4, 200349 Craiova, Romania; 2 Department of Biochemistry, Faculty of Medicine, University of Medicine and Pharmacy of Craiova, Str. Petru Rares nr. 2-4, 200349 Craiova, Romania; 3 Department of Pathophysiology, Faculty of Medicine, University of Medicine and Pharmacy of Craiova, Str. Petru Rares nr. 2-4, 200349 Craiova, Romania; 4 Department of Physiology, Faculty of Medicine, University of Medicine and Pharmacy of Craiova, Str. Petru Rares nr. 2-4, 200349 Craiova, Romania; 5 Neurosurgical Department, Clinical Hospital “Bagdasar-Arseni”, 041915 Bucharest, Romania

**Keywords:** secondary metabolites, anticancer therapy, signaling pathway, PI3K/AKT/mTOR

## Abstract

Despite the great advancements made in cancer treatment, there are still many unsatisfied aspects, such as the wide palette of side effects and the drug resistance. There is an obvious increasing scientific attention towards nature and what it can offer the human race. Natural products can be used to treat many diseases, of which some plant products are currently used to treat cancer. Plants produce secondary metabolites for their signaling mechanisms and natural defense. A variety of plant-derived products have shown promising anticancer properties in vitro and in vivo. Rather than recreating the natural production environment, ongoing studies are currently setting various strategies to significantly manipulate the quantity of anticancer molecules in plants. This review focuses on the recently studied secondary metabolite agents that have shown promising anticancer activity, outlining their potential mechanisms of action and pathways.

## 1. Introduction

After cardiovascular diseases, cancer is the second most common cause of mortality worldwide [1]. Studies show that cancer is a genetic disease, characterized by various modifications in gene expression. The “hallmarks” of cancer include, but are not limited to, the capacity of cancer cells to stimulate their growth, to resist inhibitory signals, and to evade the programmed cellular death. They have the ability to indefinitely replicate, to stimulate angiogenesis, to invade and metastasize, to skip the immune system, and to induce inflammation. Additionally, they express abnormal metabolic pathways and exert genome instability [2,3]. Conventionally, two classes of genes undergo alterations: tumor-suppressor genes and proto-oncogenes. These alterations sustain the mutated cell to bypass important checkpoints of cell division, ultimately spreading into the neighboring tissues [4]. Metastasis is initiated by angiogenesis, a process of new blood vessels’ formation from existing vessels [2]. Compared to normal cells, cancer cells exhibit chromosomal abnormalities, nuclear pleomorphism, reduction of cellular gap junction, and increased motility [5]. They are resistant to growth-suppression signals and manifest resistance to apoptosis [2]. For drug development, all these properties focusing on cell growth, invasion, and progression are possible targets in the anticancer battle.

Over the years, a vast list of drugs has been synthesized with the purpose to be used in cancer prevention and cure. However, conventional therapies have shown many drawbacks, such as: adverse effects, rapid drug resistance, and a high cost of treatment, determining researchers to continuously develop more tailored strategies for cancer treatment.

In addition to the development of either new molecules, pharmaceutical forms, or novel strategies [6,7,8,9,10], researchers have also focused their attention on natural products for the management of various diseases [11,12,13,14,15,16]. Nowadays, almost 60% of the prescribed anticancer drugs are derivatives of plant metabolites. Vincristine and Vinblastine (vinca alkaloids extracted from *Catharanthus roseus* and *Apocynaceae*), Paclitaxel (diterpenoid compound derived from *Taxus brevifolia*), Etoposide (semisynthetic podophyllotoxin extracted from *Podophyllum peltatum*), and Camptothecin (alkaloid derived from *Camptotheca acuminata*) are just a few examples of plant-derived anticancer products [17].

Nevertheless, even if they are sometimes preferred by patients due to having fewer side effects, a lower chance of developing resistance, and their affordability, various plant products have demonstrated an increased toxicity [18]. One reliable option is the combinatory therapy based on both natural and synthetic drugs.

It is well-known that the plant secondary metabolites’ synthesis using tissue cultures is not dependent on environmental conditions [19], and therefore this strategy offers a unique opportunity for large-scale production of anticancer agents.

Despite the many advantages, the use of plant secondary metabolites also has many drawbacks related to the production stage, such as the non-availability of products throughout the year [20], but also related to their pharmacology. For example, plant secondary metabolites act on many cellular pathways and on various receptors, so mono-activity is unlikely to be expected, unless chemical modifications are performed [21]. In addition, chemical instability and poor bioavailability may represent true disadvantages of secondary metabolites. To be able to measure the ratio of risk/benefit, the following outcome parameters may be considered: mortality, toxicity, pain, quality of life, ease of medication use, and costs.

As the literature on plant secondary metabolites and cancer is huge, we will take the liberty to focus on the most recent reported data and discuss emerging and interesting results in the field. Since plant pigments are able to change their structure depending on the pH [21], they are an interesting class of potential therapeutic agents for cancer therapy, which is well-known to be related to the characteristics of the tumor environment. This review aims to provide updated information regarding plant secondary metabolites with anticancer activity, and their regulated biological pathways. Nevertheless, considering the large plant metabolite resources, it seems that little has been exploited so far.

## 2. Plant Metabolites: General Overview

In recent years, a more thorough understanding of plant metabolism has led to a detailed characterization of the three main categories: primary metabolites, secondary metabolites, and plant hormones. Plants can synthesize many secondary metabolites as a response to biotic stress, or for various physiological tasks [22].

Since they can trigger pharmacological effects in humans, secondary metabolites are more and more often the focus of pharmaceutical industries [23]. Based on their applications, apart from medical uses, secondary plant metabolites can be used in other different areas, such as foods, agriculture, textile, and leather industries [23].

Based on the chemical structures, secondary plant metabolites can be divided into several classes, including phenolics (tannins, flavonoids, coumarins, chromones, xanthones, stilbenes, and lignans), alkaloids, saponins, terpenes, lipids, and carbohydrates [24]. Furthermore, based on the biosynthetic pathway, three main classes of plant metabolites may be distinguished: phenolic substances, terpenes and steroids, and nitrogen-containing substances [24].

Many alkaloid salts are used in medicine, including antitumoral vinblastine, analgesics morphine and codeine, muscle relaxant tubocurarine, antipyretic and antimalarial quinine, antiarrhythmic ajmalicine, sedative action scopolamine, antibiotic sanguinarine, gout suppressant colchicine, and reserpine used in the high blood pressure treatment. Currently, molecules derived from *Actinobacteria* are used as anticancer agents, such as actinomycin D, anthracyclines (doxorubicin, epirubicin, daunorubicin, pirarubicin, and valrubicin), mitosanes (mitomycin C), bleomycin, anthracenones (streptozotocin, mithramycin, and pentostatin), taxol, enediynes (calicheamicin), and epothilones [25].

It is worth noting that a special class is represented by the colored secondary metabolites. Due to the possible harmful effects of synthetic dyes [26], increasing development of naturally derived pigments has proven to be the main objective of many researchers. The same as synthetic dyes, colored secondary metabolites have many biological activities as antimicrobial, antioxidant, and anticancer agents. Data indicate the cancer cell-specific cytotoxic effect of many phyto-secondary metabolites.

## 3. Plant Colored Secondary Metabolites of Interest for Oncology Field

The cancer metabolism signaling pathways’ complexity, together with the toxicity, resistance, and high costs of conventional drugs, altogether determined researchers to create novel multi-target agents. Plant-derived secondary metabolites proved to be hopeful anticancer candidates able to target multiple cancer metabolism dysregulated, cross-linked pathways [23,25].

Over the last decades, colored secondary metabolites have been investigated regarding their antitumorigenic efficacy [27,28]. It has been proven that they interfere with various cell signaling pathways, such as the cell cycle pathway, survival, and cell death [29,30]. Plant colored (phytopigments) secondary metabolites have proven to increase apoptotic proteins (fatty acid synthase (Fas), BCL-2-associated X protein (Bax), Bak, Bim, and Ubiquitin C-terminal hydrolase L1 (Uch-L1)) and endoplasmic reticulum stress proteins, such as protein kinase RNA-like ER kinase (PERK), GRP78, and CCAAT-enhancer-binding protein homologous protein (CHOP). They also decreased antiapoptotic proteins (survivin, Bcl-2, Bcl-XL, inhibitor of apoptosis-1/2 (IAP-1/2), Mcl-1, and tumor necrosis factor (TNF) receptor-associated factor 1 (TRAF-1)) and inflammatory agents (cyclooxygenase-2 (COX-2), 12-lipoxygenase (12-LOX), and interleukin 6/8 (IL-6/8)). Some of the reactive oxygen species (ROS), cell cycle, or cell survival proteins’ expressions were either stimulated or inhibited. For example, plant dye secondary metabolites increased the levels of nicotinamide adenine dinucleotide phosphate (NADPH), nuclear factor erythroid 2-related factor 2 (Nrf2), lactate dehydrogenase (LDH), mitogen-activated protein kinase (MAPK), Src homology phosphatase-1 (SHP1), p53, p38, and p21, but decreased the levels of phosphoinositide 3-kinases (PI3K), nuclear factor kappa B (NF-kB), mammalian target of rapamycin (mTOR), janus kinase (JAK), extracellular signal-regulated kinase (ERK), protein kinase B (Akt), hypoxia-inducible factor-1α (HIF-1α), signal transducer and activator of transcription 3 (STAT-3), Src, glutathione (GSH), thioredoxin 1 (Trx1), cyclin D/E, and cyclin-dependent kinase 2/4 (Cdk-2/4) [31].

**Figure 1:**
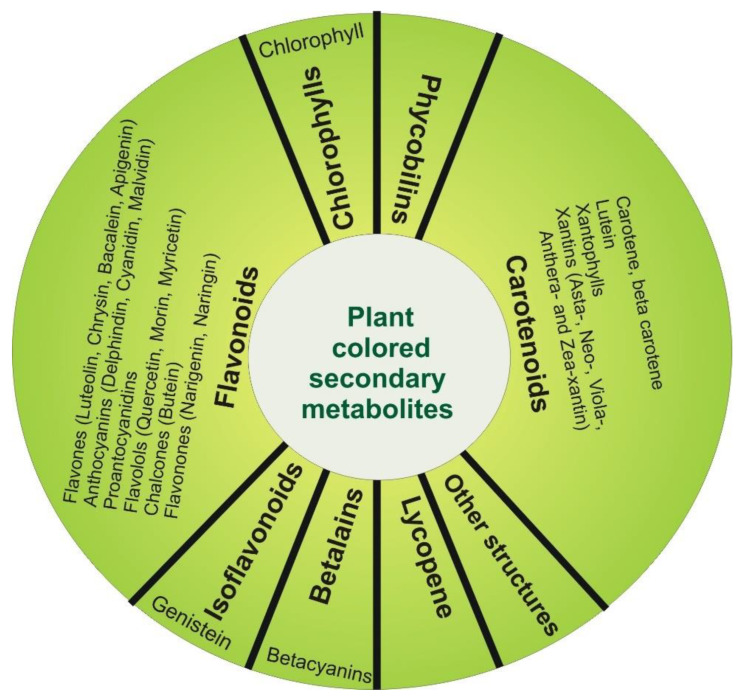
Colored secondary metabolites with anticancer effects.

With a long history of use by humans, plant pigments are very diverse. They are generally divided into: chlorophylls, phycobilins, flavonoids, isoflavonoids, carotenoids, betalains, and lycopenes (Figure 1).

Phenolic compounds are secondary metabolites of plants, usually divided into non-flavonoids (without color) and flavonoids (colored compounds as: flavonols, flavones, and anthocyanin). Phenolic compounds showed anticancer properties by targeting almost all the aforementioned cross-linked pathways. Importantly, phenolic compounds target the dysregulated pharmacological signaling pathways such as PI3K/Akt/mTOR/HIF-1α [32], Ras/Raf/MEK/ERK/MAPK [33], and JNK [34]. Additionally, they target both intrinsic and extrinsic apoptotic pathways [35]. Moreover, phenolic molecules target wingless/integrated (Wnt) and STAT3 and block inflammatory mediators (TNF-α, COX-2, and IL-6) [36].

Anthocyanins are colored water-soluble flavonoids (phenolic compounds) that color the plants in a pH-dependent manner [37]. Although they are non-essential nutrients, anthocyanins may promote health and protection against chronic diseases [38].

Anthocyanins demonstrated encouraging anticancer effects by targeting oxidative stress (NF-kB/Nrf2), inflammation (PI3K/Akt/mTOR), and apoptotic signaling pathways (Bax/Bcl-2/caspases) [39]. A limited number of studies highlighted the anticancer properties of anthocyanins but did not pay attention to the novel delivery systems [40].

Apigenin is another flavonoid pigment reported to suppress human cancers *both* in vitro and in vivo. It induces cell apoptosis and cell cycle arrest, inhibits cell migration and invasion, and stimulates the immune response by modulating signaling pathways such as the MAPK/ERK, PI3K/AKT, JAK/STAT, Wnt/β-catenin, and NF-kB pathways [41,42,43,44].

Another two flavonoid pigments present in grapefruit, naringin and naringenin, were very effective in vitro and in vivo against various cancers, including colon, breast, lung, glioblastoma, ovarian, and cervical cancers. The mechanism consisted in the initiation of apoptosis, inhibition of proliferation, suppression of metabolic activity, detoxification of carcinogens, antioxidative effect, and inactivation of oncogenes [45].

Curcumin, its derivatives, and various curcumin-based nano-systems have also been studied for the antineoplastic activity [46,47]. Curcumin alone and combined with thalidomide reduces the expression of STAT3 and Bcl-xL, leading to apoptosis in acute myeloid leukemia cell lines [48]. Curcumin upregulates the death receptors’ expression [49]. Furthermore, curcumin can downregulate intracellular transcription factors including activator protein 1 (AP-1), NF-kB, COX-2, matrix metalloproteinase-9 (MMP-9), and nitric oxide synthase (NOS). Additionally, curcumin has been found to decrease the uptake of glucose and the production of lactate (Warburg effect) by downregulating the pyruvate kinase M2 (PKM2) in cancer cell lines [50]. It is important to mention that curcumin had to be administered in very high doses to exert any effect. A major target for the anti-inflammatory effect of curcumin is the peroxisome proliferator-activated receptor gamma (PPARγ) [51,52,53].

Chlorophyll, the most abundant pigment in the world, has been reported to have a strong antineoplastic effect, with increased synergism combined with conventional anticancer drugs such as doxorubicin [54,55].

Furthermore, an important class of colored secondary metabolites is represented by carotenoids such as Beta-carotene and lycopene. Essential for both plants and animals (immune response regulators, inductors of apoptosis, inhibitors of cancer cell propagation, and suppressors of angiogenesis), carotenoids cannot be synthesized in animals. Other plant pigments such as lutein, violaxanthin, astaxanthin, and antheraxanthin have been reported to have good antioxidative and cell-protective effects [56,57,58,59].

Terpenoids are secondary metabolites that have also been found to target the main cancer dysregulated pathways. They target apoptotic pathways and PI3K/Akt/mTOR/MAPK [60].

Betalains are water-soluble colorants present in plant vacuoles of *Caryophyllales* and in mushrooms *Hygrocybe*, *Hygrophorus*, and *Amanita*. Many experiments have demonstrated betalains’ cytotoxicity in various cancer cell lines [61] by suppressing NF-kB and Akt activation [62] and by increasing oxidative stress-mediated apoptosis [63,64].

## 4. Signaling Molecules and Biological Pathways Targeted by Plant Secondary Metabolites in Cancer

**Figure 2:**
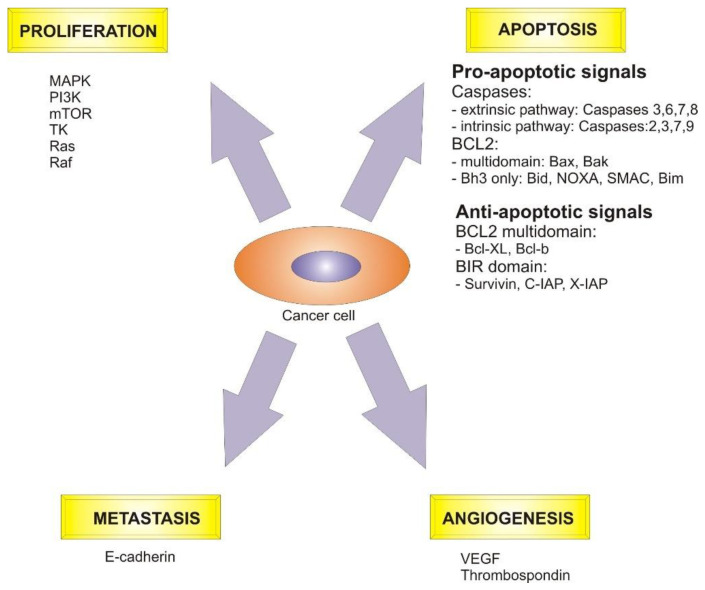
Critical signaling pathways and mediators involved in the cancer cell regulation. Abbreviations: MAPK—mitogen-activated protein kinase, PI3K—phosphoinositide 3-kinases, mTOR—mammalian target of rapamycin, TK—tyrosine kinases, Ras—rat sarcoma virus proteins, Raf—rapidly accelerated fibrosarcoma protein, SMAC—soluble membrane attack complex, C-IAP and X-IAP—inhibitors of apoptosis, VEGF—vascular endothelial growth factor.

Both cancer formation and cancer development are very important steps in cell over-proliferation. Several critical actors play key roles in these stages, such as apoptosis mediators, cell cycle control agents, and death receptors (Figure 2).

**Figure 3:**
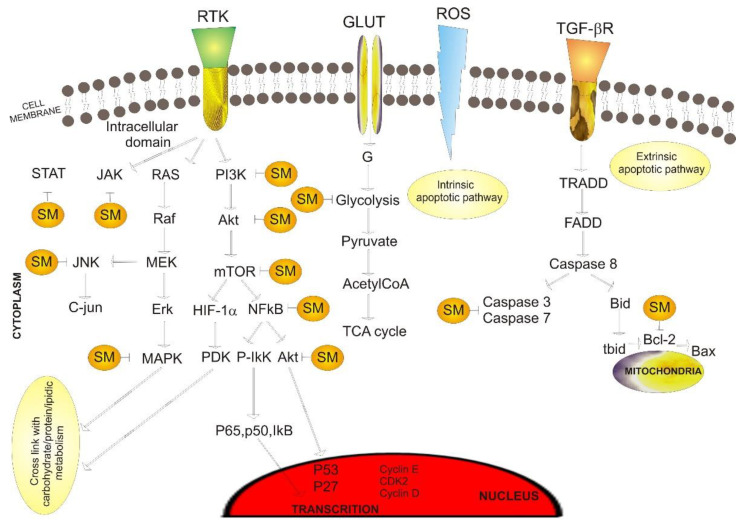
Secondary metabolites’ influence on signaling mediators of various pathways. Abbreviations: SM—secondary metabolite, MAPK—mitogen-activated protein kinase, PI3K—phosphoinositide 3-kinases, mTOR—mammalian target of rapamycin, RTK—receptor tyrosine kinases, RAS—rat sarcoma virus proteins, Raf—rapidly accelerated fibrosarcoma protein, GLUT—glucose transporter, ROS—reactive oxygen species, TGF-βR—transforming growth factor beta, JAK—janus kinase, JNK—c-Jun N-terminal kinase, MEK—mitogen-activated protein kinase kinase, Erk—extracellular signal-regulated kinase, Akt—protein kinase B, HIF-1α—hypoxia-inducible factor-1 α, NFkB—nuclear factor kappa B, PDK—pyruvate dehydrogenase kinase, G—glucose, TRADD—TNFR1-associated death domain protein, FADD—Fas-associated death domain, CDK2—cyclin-dependent kinase 2, IkB—IkappaB kinase, TCA cycle—tricarboxylic acid cycle. Point arrow—activation, block arrow—inhibition.

As previously mentioned, it has been proven that secondary metabolites can affect the signaling mediators of various pathways (apoptosis, proliferation, oxidative, and inflammatory pathways), thereby stopping cancer progression (Figure 3).

Accordingly, valuable therapeutic targets in oncology must be mentioned, such as: COX-2, JAK/STAT3, inducible nitric oxide synthase (iNOS), MMPs, colony-stimulating factors (CSFs), and growth factor receptors such as receptor tyrosine kinases (RTKs) [65].

### 4.1. Apoptosis Pathway

Apoptosis (programmed cell death) is a signaling pathway cross-linked with cancer metabolism. There are two pathways responsible for initiating apoptosis, involving the intrinsic (death receptor) and extrinsic (mitochondrial) apoptotic pathways.

#### 4.1.1. Caspases-Regulated Phases

Importantly, apoptosis occurs in three caspases-regulated phases: initiation, execution, and engulfment. Caspases 2, 8, 9, and 10 have hallmark roles in the initiation step, and caspases 3, 6, and 7 are involved in the execution phase. A caspase activation initiates procaspases, starting the apoptosis process [66].

The intrinsic pathway involves the formation of apoptotic protease-activating factor (Apaf-1) and procaspase-9, causing the release of cytochrome C and Bcl-2 family proteins (Bcl-2, Bak, Bcl-xL, and Bax) with mitochondrial membrane permeabilization [67].

The extrinsic pathway is initiated by binding the death ligands (TNF-α, Fas ligand (FasL), and TNF-related apoptosis-inducing ligand (TRAIL), activate Fas-associated death domain (FADD) and procaspase-8/10) to the TNF receptor superfamily. This causes hyperactivation of the downstream pathway of caspases 3/6/9 (caspase 3, 6, and 9) [68].

Additionally, cellular inhibitors of apoptosis proteins (cIAPs), NF-kB, and p53 are attenuating the apoptotic signaling pathways. In metastatic cells, tyrosine kinase B (TKB) activates PI3K/Akt to increase apoptosis resistance. Additionally, the JAK/STAT pathway is also involved in the resistance to apoptosis and transforming growth factor-beta (TGF-β), c-Jun N-terminal kinases (JNK), and MMP are other signaling mediators involved in metastasis activation [69].

In cancer, the suppression of the apoptotic mechanism through various mechanisms leads to the loss of homeostasis. Cytotoxic agents kill tumor cells by activating the mechanism of apoptosis. Instead, defects in apoptosis signaling pathways contribute to tumor resistance [70]. Many natural agents have been shown to induce the apoptotic pathway in cancer both in vitro and in vivo [71]. It has been proven that plant metabolites may directly activate the initiator caspases or the execution caspases [72].

Plant extracts with anticancer effects may increase the expression of death receptors on tumor cells. The extrinsic pathway starts when various death receptors are expressed on the cell surface. There are six mammalian death receptors: TNF receptor 1 (TNFR1), death receptor 3/6 (DR 3/6), TNF-related apoptosis-inducing ligand receptor (TRAILR 1/2 or DR 4/5), and Fas receptor (FASR). Luteolin upregulated death receptors and death receptor downstream factors such as TRAIL 2 in human liver cancer cells [73].

Moreover, the intrinsic pathway is based on mitochondrial initiated events, such as the increase of mitochondrial membrane permeability and the release of proapoptotic proteins [74].

Quercetin is a polyphenolic flavonoid abundantly found in red grapes, apples, raspberries, honey, cherries, and green leafy vegetables, with anticancer effects in a multi-targeted manner [75]. Quercetin can induce apoptosis by decreasing the mitochondrial membrane potential in human epidermoid carcinoma cells KB and KBv200 [76,77]. The mechanism of apoptosis was linked to the release of cytochrome C, cleavage of poly (ADP-ribose) polymerase, and activation of caspase 9 and 3. The apoptosis was not related to the regulation of B-cell lymphoma 2 (Bcl-2) or BCL2-associated X protein (Bax) in KB and KBv200 cells [76,77]. In addition, quercetin has been proven to induce apoptosis by inhibiting the pathway PI3K–Akt-mTOR in Dalton’s lymphoma ascite (DLA) cells [78].

Responsible for the aroma and color of plants, flavonoids are secondary plant metabolites widely studied as therapeutic agents due to their properties such as: antioxidant, anti-inflammatory, anti-allergic, antibacterial, antiviral, and anticancer effects [79]. For example, luteolin, a flavonoid present in plants such as parsley, carrots, cabbages, and celery, activates caspases 3, 8, 9, and 10, inducing apoptosis in vitro [31]. Another flavonoid, quercetin, induced apoptosis by activating caspases 3 and 7 [31]. Phytic acids (inositol hexaphosphate, IP6) from rice bran enhance the expression of caspases 3 and 8 in liver and colon cancer cells [80], and furthermore potentiate oxaliplatin effects in colorectal cancer [81].

Other proteins such as caspase-activated DNase (CAD) and inducing factor (AIF) can initiate apoptosis in a caspase-nondependent manner. These proteins cause DNA fragmentation in the nucleus, initiating the apoptotic pathway [82]. Curcumin has been found to induce the caspase 3-dependent apoptotic pathway, but inhibited DNA fragmentation factor 40/caspase-activated DNase endonuclease in human Jurkat cells [83]. In a recent study, curcumin dramatically inhibited the cell growth of a human cell line (HaCaT) and stopped the cells in the G2/M phase. The apoptosis-inducing factor (AIF) was released from the mitochondria to the nucleus, where it increased its expression in the nucleus. The ratio of Bax to Bcl-2 was also increased, initiating the caspase 3 activation. These data suggest that the apoptosis induced by curcumin occurs through the caspase-dependent and caspase-independent pathways [84].

Furthermore, the final stage of apoptosis is the phagocytes engulfment of the apoptotic cell. The TNF receptor FAS, caspase 8, and caspase 3 externalize phosphatidylserine on the apoptotic cells’ surface, mediating the attraction of phagocytes [85]. Although plant secondary metabolites have shown outstanding anticancer effects by activating the initiation phase, extensive research needs to be performed to determine their effect on the execution and engulfment phases.

As previously mentioned, secondary metabolites can stop cancer progression by affecting the signaling mediators of the oxidative and inflammatory signaling pathways.

#### 4.1.2. NF-kB Pathway

NF-kB is the only signaling molecule which governs both pro- and anti-inflammatory, and pro- and anti-apoptotic responses, because of the canonical and non-canonical parts of the NF-kB signaling pathway. Altogether, these pathways regulate cancer-related inflammation, neoplasia, hyperplasia, and metastasis [86]. NF-kB overexpression induces cell proliferation, and it has been reported in various cancers, including breast and pancreatic cancer, multiple myeloma, head and neck squamous cell carcinoma, and melanoma [87]. Furthermore, NF-kB activation has been proven to be regulated by phosphorylation of high basal IκBα kinase (IkB or IKK) and NF-kB p65 subunit. Linked to this mechanism, various phyto-colored molecules inhibited NF-kB by suppressing the IKK phosphorylation and prevented its localization into the nucleus. For example, apigenin suppressed the expression of NF-kB and NF-kB via the IKK pathway in mice with transgenic adenocarcinoma of the mouse prostate [43].

Chrysin is a flavone found in *Passiflora caerulea* and *Passiflora incarnata*, and in *Oroxylum indicum*. Recent studies have shown that chrysin inhibits NF-kB-dependent C-C motif chemokine ligand 5 (CCL5) transcription by targeting IκB kinase and partially decreases the inflammatory responses [88].

Carotenoid tetraterpenoids have the ability to affect the NF-kB signaling pathway. For example, lutein is responsible for IκBα degradation and inhibits the p65 subunit’s nuclear localization [89]. Lycopene is another carotenoid, extremely potent as an antioxidant, that exhibits anti-proliferative and anticancer properties. Lycopene and β-carotene inhibited the IκB phosphorylation, NF-kB transcriptional activity, and suppressed the TNF-induced nuclear translocation of the p65 subunit in vitro [86,90,91].

#### 4.1.3. ROS

Another main factor that activates signaling pathways to cancer initiation is the oxidative stress, a process due to the imbalance between the production and elimination of free radicals or ROS. In order to reach a balance, cells release several antioxidants, including superoxide dismutase (SOD), catalase (CAT), and GSH. In this situation, the cells activate the survival or proliferation signaling pathways: PI3K/Akt, ERK/MAPK, NF-kB, and AP-1 [92]. Furthermore, ROS also activates other regulator molecules such as p53, HIF-1α, and STAT3, which can also play the role of upstream mediators of inflammation. NF-kB, PI3K/Akt/mTOR, Nrf2, and nuclear factor of activated T cells (NFAT) have critical roles as inflammatory signaling mediators [93]. PI3K/Akt/mTOR dysregulation represents a pivotal signaling pathway, which initiates other downstream pathways and mediators.

Many research works have shown that plant color compounds have the ability to stimulate pro-apoptotic effects through various processes, such as overproduction of ROS, loss of mitochondrial membrane potential, and modulation of apoptotic proteins such as Bax/Bcl-2. The stress-causing ROS activity can be stimulated into the cancer cells by various plant coloring compounds, such as juglone, bixin, curcumin, quercetin, shikonin, beta-carotene, rhein, morin, danthron, and apigenin.

For example, Juglone, a dark reddish brown color secondary metabolite from *Juglans nigra*, and its derivatives induce ROS accumulation in cancer cells, causing DNA fragmentation and GSH [94].

Bixin, another secondary metabolite found in the seeds of *Bixa orellana*, and curcumin from *Curcuma longa*, both stimulate ROS and inhibit the TrxR/Trx pathway [95,96].

Additionally, Rhein and Baicalein induce ROS, resulting in the endoplasmic reticulum (ER) stress, which further activates caspase 12, promoting apoptosis [97]. Wang et al. reported that luteolin induces ROS via activating ER stress in GB cell lines and in nude mice in vivo [98].

Furthermore, Nrf2 is involved in a Keap1-Nrf2/EpRE/ARE pathway, known to regulate various antioxidant genes [99]. Secondary metabolites such as carotenoids and apocarotenoids have been proven to be involved in the upregulation of Nrf2 in transiently transfected cancer cells [100]. Rottlerin, a polyphenolic compound from *Mallotus philippinensis*, inhibits ROS formation and prevents the activation of NF-B in breast MCF-7 and colon HT-29 cancer cell lines [71,101].

### 4.2. Cell Survival Pathways

#### 4.2.1. PI3K/Akt Pathway

The hallmark of cancer is the uncontrolled proliferation rate of cells. Alterations occur as modifications in enzymatic processes, and metabolic and signaling pathways.

The phosphoinositide 3-kinase (PI3K)/Akt is a critical pathway for tumor development and progression. It is activated in tumor cells by mutations in gene encrypting involved in metabolism, proliferation, motility, and survival, and in normal cells it is inhibited by the tumor suppressor phosphatase and tensin homolog (PTEN). The cytokines bind to the specific receptor activating Ras, and further, PI3K. The downstream consists in PI3K phosphorylation, leading to Akt (protein B kinase) activation, which contributes to the antiapoptotic protein Bcl-XL’s restoration. Akt also modulates NF-kB and the cell cycle pathway by activating IKB kinase (IKK) and by preventing cyclin D1 degradation [32].

It has been proven that colored secondary metabolites such as flavones and flavonoids have the ability to inhibit the cell survival PI3K/Akt pathway [102]. For example, genistein, the primary secondary metabolite from *Trifolium* species, and curcumin from *Curcuma longa*, have been demonstrated to hinder the cancer uncontrolled cell proliferation by targeting the PI3K/Akt pathway [103,104]. Ji et al. reported that quercetin inhibits the Akt/mTOR pathway and initiates apoptosis by stimulating autophagy, in a study using hepatic cancer cells [105]. Moreover, cyanidin, a phytopigment found in many red berries, has been proven to inhibit hepatic cancer cell migration and to enhance the effects of oxaliplatin via suppressing the PI3K/Akt pathway [106].

Furthermore, another pathway involved in cell growth regulation and proliferation is the mammalian target of rapamycin (mTOR). Activated by AKT, the mTOR pathway can represent an effective target to treat various types of cancer. Many studies involving phytocompounds have reported their inhibitory effect on the mTOR pathway. For example, beta-carotene, myricetin, and quercetin from *Ajwa* dates’ pulp induced apoptosis by inhibiting the AKT/mTOR pathway and by modulating Bcl-2 family proteins in human breast cancer [107]. Additionally, curcumin has been shown to inhibit the progression of human gastric cancer by regulating the mTOR pathway [108]. Moreover, oroxylin A is an O-methylated flavone found in *Scutellaria* species which has been shown to inhibit the proliferation by suppressing the Akt/ERK activation and the mTOR and STAT3 phosphorylation, inducing autophagy in glioma cells [109].

It is known that the mTOR pathway upregulates the HIF1 expression, which further upregulates the enzymes involved in glycolysis and glucose transporters. Although HIF-1 signaling occupies a central role in normal cells as an orchestrator of the adaptive response of glycolysis to hypoxia, interestingly, the loss of HIF-1 in cancer cells does not perturb the glycolytic phenotype [110]. HIF1 also activates the expression of various gene-encoding growth factors, such as vascular endothelial growth factor (VEGF), promoting angiogenesis [111]. Quercetin has been proven to inhibit the HIF-1 transcriptional activity in colon cancer cell lines. Moreover, the decrease of HIF-1 activity was linked to the AMP-activated protein kinase (AMPK) inhibition, leading to quercetin-induced apoptosis in the hypoxic environment in vitro. In addition, the in vivo administration of quercetin decreased tumor progression in a xenograft model [112].

Baicalein, a flavone secondary metabolite isolated from *Scutellaria baicalensis*, expresses various beneficial properties, including anticancer effects demonstrated in many studies [113]. For example, baicalein sustained apoptosis and cell cycle arrest, decreasing tumor growth and proliferation, in orthotopic gliomas in mice. Interestingly, it reduced HIF-1α protein expression in U87 glioma cell lines [114]. Additionally, baicalein suppression of HIF-1α contributed to cell viability reduction and enhanced cisplatin efficacy in ovarian cancer cell lines [115]. Fang and coworkers reported that luteolin inhibited angiogenesis by downregulating the HIF-1α and STAT3 signaling pathway under hypoxic conditions in the mouse macrophage RAW264 cell line [116]. Furthermore, flavonoids have been shown to affect HIF-1 transcriptional activity by impairing the MAPK pathway, inhibiting nuclear accumulation and phosphorylation of HIF-1α in cervical cancer cells [117].

p53 is a tumor suppressor, described as “the guardian of the genome” in normal cells because it detects DNA damage and stops the cycle for repair or leads the cell into apoptosis by activating the caspase pathway. However, in cancer cells, p53 is downregulated. Additionally, in cancer, p53 activates the enzyme hexokinase 2, regulating the glycolytic phenotype [118]. Several plant secondary metabolites induce apoptosis via the p53-dependent pathway. Anthocyanins inhibit cancer proliferation by targeting p53, p21, and cyclin A and D [118].

#### 4.2.2. MAPK Pathways

The mitogen-activated protein kinase pathways are involved in the regulation of cell growth, differentiation, migration, and apoptosis, for which they are frequently used as a target for various cancer therapies. To date, three groups of MAPKs have been reported in mammals: ERKs (extracellular signal-regulated kinases), JNKs (Jun amino-terminal kinases), and p38/SAPKs (stress-activated protein kinases) [119].

Zhang and coworkers reported that MAPK pathway signaling follows a cascade, in which MAP3K activates MAP2K, which further activates p38, ERK, or JNK, resulting in activation of NF-kB [120].

It is worth noting that the MAPK pathway may also activate both growth- and apoptotic-inducing genes (p53, c-myc, c-jun, ATF2, Bcl-2, Bcl-XL, and E1K-1) [121].

*ERK Pathway*

Goel et al. reported that coumarin induced apoptosis via inhibition of the ERK/MAPK pathway in a lung adenocarcinoma cell line, showing that p21, Cox-2, and p53 have been downregulated and c-Myc has been upregulated [122].

In another study, quercetin induced apoptosis by inhibiting the ERK1/2 activities and by enhancing the caspases’ activation in human leukemia cells [123]. Along with luteolin, quercetin showed important antiproliferative and apoptosis-inducing effects by targeting B-RAF- and K-RAS-activating mutations in gastric cancer cell lines by decreasing the ERK phosphorylation [124]. Furthermore, studies on astaxanthin showed that it induced apoptosis and increased the activity of antioxidant enzymes in a colorectal cancer cell line through inactivation of ERK/Akt [125].

*JNK Signaling Pathway*

The JNK involvement has been reported by Feng et al., who proved that the combination of luteolin and sorafenib killed human hepatocellular carcinoma cells via JNK activation and apoptosis facilitation [126]. Furthermore, the findings of the study performed by Nazim and coworkers also indicated that luteolin enhanced TRAIL-initiated apoptosis, and suggested that these effects were mediated by JNK-mediated DR5 expression and autophagy in human liver cancer cells [73]. The results from a recent study on mice demonstrated that the combination of luteolin and oxaliplatin synergistically inhibited colorectal tumor growth by potentiating apoptosis and by inhibiting proliferation, probably through an AMPK-associated mechanism. These findings indicate that a diet rich in luteolin could improve the efficacy of oxaliplatin in colorectal cancer treatment [127].

*p38 Pathway*

Curcumin, the phenolic secondary metabolite from *Curcuma longa*, is known to have an antineoplastic activity compound. It was reported that curcumin can inhibit the phosphorylated p38 expression in the malignant gliomas cell line. Furthermore, MicroRNA-378 can counteract the inhibitory effect of p38 by increasing its phosphorylation and by enhancing the cells’ sensitivity to curcumin [128]. A curcumin metabolite—tetrahydrocurcumin—showed anticancer activities by inducing mitochondrial apoptosis and cell cycle arrest via activation of p38-MAPK in human breast cancer cells [129]. However, curcumin has a limited clinical application due to its low absorption, poor bioavailability, and rapid metabolism. To overcome these drawbacks, curcumin-based derivatives are currently being developed [130,131].

#### 4.2.3. JAK/STAT Pathway

Signal transducer and activator of transcription (STAT) signaling proteins can be activated by various factors, such as cytokines, interferons, and growth factors, and furthermore, they are substrates for Trk of the Jak and Src53 families. Dysregulated STAT activation determines increased angiogenesis and increased tumor survival [132].

Morin, a flavonol secondary metabolite present in plants from the *Rosaceae*, *Moraceae*, and *Fagaceae* families, has been proven to inhibit the STAT activity by JAK, SHP1, and Src kinase activation [133]. Indirubin, another morin plant colorant, also inhibits STAT activity in prostate and human breast cancer cell lines [134].

### 4.3. Cell Cycle Mechanism

Apart from cancer formation, cancer development is also an essential step in cellular over-proliferation. Death receptors, cell cycle control, and apoptosis mediators play critical roles in this stage.

All living organisms reproduce by the cell cycle process, initiated by cyclin-dependent kinase (CDK) cascade activation. G1/S and G2 are two important checkpoints in the cell cycle pathway. In all normal cells, cyclin D1 and E bind to cyclin-dependent kinases (Cdk4 and Cdk2) that phosphorylate retinoblastoma (Rb), releasing the group of genes that encode the transcription factor in eukaryotes (E2F). Metabolites such as quercetin, curcumin, resveratrol, flavopiridol, and genistein have been reported to inhibit CDKs [135,136,137]. Most of the plant-derived colorants influence and induce cell cycle arrest via DNA damage, causing apoptosis.

Topoisomerase II is an important enzyme involved in DNA replication during cellular division. It regulates the DNA downstream negative supercoiling of the replication fork. Topoisomerase catalytic inhibitors decrease catalytic turnover of topoisomerase. A few well-known examples of topoisomerase II poisons are podophyllotoxin derivatives (etoposide and teniposide), fluoroquinolones, and doxorubicin. Additionally, secondary metabolites such as curcumin have been reported to act as catalytic inhibitors and topoisomerase poisons [138]. It must be mentioned that topoisomerase II poisons are regularly consumed by humans in their daily diet, such as bioflavonoids (flavones, isoflavones, and flavonols), catechols, catechins, isothiocyanates, and quinones. For example, genistein acts as an interfacial poison, and it is highly active against both topoisomerase IIα and β and shows an efficacy and potency against the enzymes comparable with that of etoposide [139]. Although the parent compound displayed no activity against topoisomerase II, the curcumin quinone oxidation intermediates proved to be potent poisons by disrupting the cell cycle mechanism [140].

### 4.4. Angiogenesis

Angiogenesis is another critical cross-linked pathway from the cancer progression process. It involves activation, proliferation, and migration of cells with the purpose to form novel blood vessels. Tumor cells create new vessels from existing ones by liberating various factors that induce angiogenesis in a direct or indirect manner (angiogenic switch), followed by extracellular matrix degradation and endothelial migration. There are many signaling pathways, mediators, and downstream signaling proteins reported in angiogenesis-induced cancer: platelet-derived growth factor (PDGF), VEGF, fibroblast growth factors (FGF), hepatocyte growth factor (HGF), TKs, PI3K, MAPK, mTOR, Ras, and Raf [71,141,142,143,144].

Many factors such as hypoxia and oncogenes (Ras and Myc) signals upregulate the genes encoding VEGF [141]. The vascular-disrupting agents can be classified as: ligand-directed agents and small-molecule agents. Several plant secondary metabolites such as tannins, flavonoids, and triterpenoids have been proven to inhibit angiogenesis in cancer cells [71].

One of the most studied classes of secondary metabolites is represented by the anthocyanins, which proved multiple biological activities (neuroprotection, cardio-protection, antidiabetic, anti-obesity, and anticancer effects) [145].

VEGF-induced angiogenesis was markedly inhibited by anthocyanins and flavonoid glycosides from black raspberry extract in two organ-specific primary cells (human intestinal microvascular endothelial cells and human esophageal microvascular endothelial cells) [146]. Moreover, berry anthocyanins significantly inhibited both H_2_O_2_- and TNF-alpha-induced VEGF expression in human keratinocytes [147].

Naringenin, a plant secondary metabolite found in tomatoes and oranges, showed an inhibitory effect on the VEGF/KDR signaling pathway in a malignant melanoma cell line [148].

Delphinidin is a purple-colored plant pigment, which exerts a multitude of useful biological activities by complex and distinct mechanisms. As an antineoplastic agent, it has been reported to inhibit angiogenesis through the suppression of VEGF and HIF-1α expression in lung cancer cells. Researchers reported that delphinidin inhibited HIF-1 and ERK, P13K/Akt, and mTOR/p70S6K signaling pathways [149].

Turmeric, recognized for its medicinal effects, has received interest from the scientific world as it is the major source of curcumin. Curcumin targets multiple signaling molecules, demonstrating activity at the cellular level. It directly inhibits angiogenesis both in vitro and in vivo and it can downregulate some pro-angiogenesis factors [150]. Curcumin affects the whole angiogenesis process by downregulating transcription factors such as NF-kB and pro-angiogenesis factors such as VEGF, MMPs, and bFGF [151]. Despite the antiangiogenetic properties, it has been proven that curcumin also possess a pro-angiogenetic effect. A study showed that a pretreatment with curcumin may augment the adipose-derived stem cells’ production of VEGF, contributing to new vessels’ formation [152].

A common flavonoid that can be found in many types of plants, luteolin has been shown to exert antiangiogenetic functions. It has been demonstrated to decrease the expression of VEGF, VEGFR2, HIF-1α, MMP-1, MMP-9, Notch1, and P-Akt in a gastric cell line [153].

Another natural dye compound is quercetin, a flavonol extracted from *Sophora japonica*. Quercetin inhibited malignant cell growth in leukemia, melanoma, ovarian, breast, gastric, bladder, colon, and lung cancers [19]. Its antiproliferative effect has been explained by different mechanisms’ activation of ERK-dependent COX-2/PGE, and inhibition of PI3K-AKT/PKB and IL-3/STAT3 signaling pathways [154]. It also promotes caspase 3-mediated apoptosis. Quercetin inhibited angiogenesis by abrogating VEGFR2 levels and by inhibiting PI3K/AKT, MEK/ERK, and MEK/JNK signaling pathways [155,156].

Furthermore, chrysin significantly downregulated HIF-1, VEGF, and VEGFR-2, but not VEGFR-1, gene expression both in vitro and in vivo in human umbilical endothelial cells and in chicken chorioallantoic membrane. Additionally, it suppressed the hypoxic survival and metastatic growth in a mouse breast cancer cells experiment [157,158]. Despite chrysin’s efficacy in oncology, a few major challenges hinder its clinical use. The low aqueous solubility, low oral bioavailability, physicochemical instability, and rapid metabolization led scientists to improve its properties. Nano-emulsions, nanosuspensions, nano-micelles, self-assembled nanoparticles, host–guest complexes, derivatizations, and salt formation approaches have been used, with promising results [159].

### 4.5. Metabolic Alterations

It is well-known that there are several major dysregulated pathways that create the context for cell over-proliferation. The metabolic alterations generally focus on rapid production of adenosine triphosphate (ATP), and of macromolecules necessary for cell viability. The altered metabolic pathways involve crucial elements such as oxygen, glucose, glutamine, and ATP. The Warburg effect is a modification in cancer metabolism, where the production of energy is diverted from normal oxidative phosphorylation to aerobic glycolysis [160].

Briefly, because tumor cells need a large supply of energy, they over-activate glucose transporter 1, and pyruvate is continuously produced from glucose. Further, it has two fates: it is either converted to lactate or it can suffer oxidative decarboxylation, releasing NADPH and malate in the mitochondria [161]. It has been reported that glucose transporters are upregulated in a cell-specific manner in cancer cells [162]. Related to this aspect, plant compounds have been proven to target glucose transporters. For example, in a study on breast cancer cells, naringenin inhibited the PI3K pathway, a regulator of glucose transporters, and therefore it inhibited glucose uptake [163,164,165]. Another negative regulator of glucose transporters has been proven to be baicalein. Chen et al. reported that baicalein re-sensitized the tamoxifen-resistant breast cancer cells via two mechanisms: by reducing aerobic glycolysis and by reversing mitochondrial dysfunction through inhibition of HIF-1α. Baicalein decreased glucose uptake, ATP generation, lactate production, the lactate/pyruvate ratio, and HIF-1α-targeted glycolytic genes’ expression. It also interfered with HIF-1α inhibition of mitochondrial biosynthesis. This increased the mitochondrial DNA content, restored the ROS, and thus increased the tamoxifen-induced mitochondrial apoptotic pathway [166].

LDH catalyzes lactate synthesis from pyruvate under anaerobic conditions [167]. Some plant compounds have been screened to quantify their potential in inhibiting LDH [168]. For example, galloflavin was discovered to be a potent LDH inhibitor, which decreases the viability of the hepatocellular carcinoma cell line [169].

Moreover, genistein derivatives decreased LDHA protein levels and downregulated glucose uptake, ATP generation, and lactate production in a study involving breast cancer cells [170].

As seen, plant secondary metabolites use a variety of mechanisms that interfere with the development, promotion, and progression of cancer, through modulating signaling pathways associated with proliferation, inflammation, apoptosis, autophagy, invasion, angiogenesis, and metastasis (Table 1).

Considering all this, it is worth mentioning that plant dyes are highly useful for the development of a large variety of new drugs. Since they show great promise for the future, no doubt plant secondary metabolite dyes will fulfill many needs of today’s medicine.

## 5. Combination Therapies Involving Secondary Metabolites

Inspired by nature, secondary metabolites are gaining attention for their clinical use. Evolutionarily conserved elements of plants, colored secondary metabolites have distinct advantages compared to conventional drugs. However, some inherent drawbacks need to be overcome for their clinical translation. Among them, the short half-life due to protease degradation susceptibility, the cytotoxicity towards normal cells, and the limited targeted delivery must be mentioned. Currently, for an effective concentration at the target site, secondary metabolites have been used in relatively high doses. Therefore, agents that can increase their bioavailability and specificity on the target site could be used.

On the other hand, another purpose of the combination therapy is to improve the magnitude of therapeutic responses and to reduce the acquired resistance in a patient. One major benefit of adding secondary metabolites in combination therapies is that they reduce the chance to develop drug resistance. Various combinations of secondary metabolites and chemotherapeutic agents are already in clinical phases [171,172,173].

The natural products are very interesting as they can augment the activity of chemotherapeutics or even partially reverse the multidrug resistance of well-adapted and resistant cells. If secondary metabolites are used in combination with a cytotoxic agent, they may reverse the resistance of the latter in a synergistic manner [18] and may also increase the chemotherapeutic drugs’ susceptibility. Combinatory treatments have been studied to overcome drawbacks such as toxicity, serious side effects, and drug resistance at high dosages. One such studied combination has been tested with secondary metabolites and clinically approved anticancer drugs (e.g., 5-fluorouracil, doxorubicin, irinotecan, cisplatin, and Paclitaxel). Data provided by Zhang et al. show that a curcumin pre-treatment, followed by 5-fluorouracil administration, increased the susceptibility of the chemotherapeutic agent in colon cancer cells and xenograft [174]. Another study found that the combination of docetaxel and curcumin significantly inhibited the proliferation and induced apoptosis in prostate cancer cell lines when compared to docetaxel or curcumin alone, via modulation of PI3K, p53, NF-κB, RTK, COX-2, and phospho-Akt [175]. There are several clinical studies currently ongoing that are testing the combination of curcumin and chemotherapeutic drugs. For example, curcumin in addition to folinic acid/5-fluorouracil/oxaliplatin chemotherapy represents a tolerable and safe treatment able to provide benefits to cancer patients [172].

Another colored secondary metabolite, luteolin, has been proven to sensitize cisplatin’s antitumor effect in drug-resistant ovarian cancer by inducing apoptosis and inhibiting cell migration and invasion in vitro [176]. Additionally, Zhang et al. recently showed that the combination between luteolin and lapatinib enhanced the therapeutic efficacy of the second mentioned agent via the FOXO3a/NQO1 pathway on human breast cancer cells [177]. No clinical trials are currently available regarding luteolin administration in cancer therapy.

A Phase I/II trial is ongoing regarding isoquercetin as an adjunct therapy in combination with Sunitinib in kidney cancer patients [171].

Other results published by Lim and coworkers indicated that delphinidin inactivated the PI3K/AKT and ERK1/2 mitogen-activated protein kinase signaling cascades, inhibiting the proliferation of ovarian cancer cells [178].

One recent clinical trial involving the study of the carotenoid lycopene in combination with docetaxel in cancer patients with chemotherapy-naïve prostate cancer published encouraging results. The study suggested that the combination of docetaxel and lycopene has a favorable activity in metastatic castrate-resistant prostate cancer patients [173].

Furthermore, beta-carotene has been proven to synergistically enhance the anticancer effect of 5-fluorouracil in esophageal squamous cell carcinoma both in vivo and in vitro [179]. However, a recent meta-analysis indicated that supplementation with beta-carotene had a negative effect on the lung cancer risk in smokers and asbestos industry workers [180].

In a very recent study published by Shehatta et al., the natural flavonoid baicalin enhanced the antitumor effect of 5-fluorouracil in breast cancer in an Ehrlich solid tumor mice model by inhibiting angiogenesis and tumor growth [181].

One of the main principles of worldwide researchers is to develop therapeutic agents with increased bioavailability. The idea of a tumor tissue compartment in which a secondary metabolite has the major activity is of great relevance. Addressing cells from different tumor compartments requires special drug carriers, which must be able to accumulate via the enhanced permeability and retention effect, but also to extravasate, and further to target specific populations of cells, and finally, to be internalized by them. In a study performed by Tsvetkova et al., passive and active targeting to different tumor compartments has been achieved using functionalized polymeric nanocarriers [182].

These results may represent important data to consider secondary metabolites as pivotal therapeutics for the prevention of various cancer types, including drug-resistant cancers.

## 6. Conclusions

Considering that cancer cells express dysregulated metabolism and pharmacological signaling pathways and that conventional drugs show multiple side effects, increased resistance, and high costs, particular attention must be paid to natural anticancer products, such as plant-derived secondary metabolites.

In vitro experimental studies revealed that many phyto-colorants expressed anticancer activity through various mechanisms of targeting signaling mediators in cancer metabolism, including PI3K/Akt/mTOR, Bax/Bcl-2/caspases, and NF-kB/Nrf2. Based on encouraging preliminary in vitro results, phyto-colorants play the role of attractive candidates for novel anticancer drugs.

Further knowledge of the mechanisms of phyto-colorants will help to predict related clinical relevance in the treatment of different cancers. Future detailed in vitro and in vivo experiments should be performed to investigate the signaling pathways of colored secondary metabolites, followed by consolidation with clinical studies in this research area.

## Figures and Tables

**Table 1:** Examples of colored secondary metabolites and their targets in cancer therapy.

Class	Subclass	Examples	Targets	Type of Study	References
Flavonoids	Flavones	Luteolin	JNK, Akt, Caspase 9, 8, 3 NF-kB, Bcl-2, Bax, Bad, SOD, Catalases, MAPK, Bak, Cyclin D1, Cdk 4, 6, Rb, Fas, FasL	in vitro tests	[73,80,81,98,116,125,126,153]
			Caspase 9, 8, 3, Bax, ROS	in vivo tests	[81,127]
		Chrysin	NF-kB, p21, p38, Akt, Caspase-3, TNFα, Cdk 2, 4	in vitro tests	[88,157,158]
			NF-kB, p65, MAPK	in vivo tests	[157]
		Baicalein	Akt	in vitro tests	[97,115,166]
			Ras, Raf, MAPK, Bcl-2, p53, Bax, Caspase 3, 9	in vivo tests	[97,113,114]
		Apigenin	Akt, PI3K, p53,	in vitro tests	[41,42]
			p21, NF-kB, Caspase-3	in vivo tests	[41,43,44]
	Anthocyanins	Delphinidin	PI3K, Akt, mTOR, p38, NF-kB, Bax/Bcl-2	in vitro tests	[149]
		Cyanidin	PI3K, Akt, mTOR	in vitro tests	[106]
		Malvidin	p53, p38, Akt, MAPK	in vitro tests	[39,118,146]
	Flavonols	Quercetin	Caspase-3,9, ROS, PI3K, Akt, Bad/Bax, NF-kB, p53, Bcl-2/Bax	in vitro tests	[76,77,78,107,112,154,155,156]
			Bcl-2, Caspase-3, Akt, MAPK	in vivo tests	[81,105]
		Morin	STAT3, Src, Bcl-2, Bcl-xL,	in vitro tests	[133]
		Myricetin	Akt/mTOR, Bcl-2	in vitro tests	[107]
	Flavonones	Naringenin	ERK, MAPK, STAT3, NF-kB, p65, ROS, PI3K/AKT/mTOR	in vitro tests	[148,163,164,165]
			NF-kB, ROS	in vivo tests	[165]
		Naringin	ERK, PI3K/AKT/mTOR, ROS	in vitro tests	[45]
Isoflavonoids	Genistein		Akt, NF-kB, PI3K, ROS	in vitro tests	[103,135,136,137,170]
Chlorophylls	Chlorophyll		PI3K, Akt	in vitro tests	[54]
			Wnt/β-catenin	in vivo tests	[55]
Carotenoids	Carotene and Beta-carotene		PPARγ, p21, Nrf2, Cyclin D1, Bax/Bcl-2, NF-kB, p21, ROS	in vitro tests	[86,90,91,107]
	Lutein		PI3K, Akt, NF-kB	in vivo tests	[89]
	Xanthins	Asta-, Neo-, Vola-, Anthero-, Zea-Xantin	ROS, MAPK, Akt	in vitro tests	[125]
	Lycopene		NF-kB, Cyclin D1, p27, Caspase 8, 9, Bcl-2	in vitro tests	[86,90,91]
Betalains	Beta-cyanins		Bcl-2, BAD, NF-kB, Akt Caspase 3, 8, 9,	in vitro tests	[61,62,63,64]
			NF-kB, Akt	in vivo tests	[62]
